# Inadvertent Entrapment of a Central Venous Catheter by a Purse-String Suture during Cardiopulmonary Bypass: A Case Report

**DOI:** 10.1155/2011/760426

**Published:** 2011-12-18

**Authors:** Abdorasoul Anvaripour, Forouzan Yazdanian, Mohammad-Zia Totonchi, Houshang Shahryari

**Affiliations:** ^1^Anesthesiology Department, Bushehr University of Medical Sciences, Bushehr, Iran; ^2^Anesthesiology Department, Tehran University of Medical Sciences, Tehran, Iran

## Abstract

A 65-year-old female patient with severe mitral valve stenosis plus coronary artery disease was scheduled for mitral valve replacement and 2-vessel coronary artery bypass graft (CABG) surgeries simultaneously. After a successful procedure, resistance was met on a CVC withdrawal. During postoperative fluoroscopy, fixation of the catheter at the heart was confirmed which necessitated reopening the chest, cutting the suture, and removing the catheter. When a catheter became hard to withdraw after open heart surgery, we should never withdraw it forcefully and blindly. Although rare, one should consider inadvertent entrapment of CVC by a suture as the possible cause.

## 1. Introduction

Central venous catheterization which is a routine procedure in patients undergoing open heart surgery is frequently used for hemodynamic monitoring. A variety of catheter-related complications has been reported in literature. Among them, problems encountered infections and mechanical complications are most common complications [1].

There are many reports of the complication by the central venepuncture, but there are few reports about its entrapment in an atrial sutures during open heart surgeries in the literature [2–5].

In this paper, we represent a very rare case of accidentally CVC entrapment into the purse-string suture at the venous cannulation site of cardiopulmonary bypass (CPB) during open heart surgery that necessitated reopening the chest, cutting the suture, and removing the catheter. We wish to highlight concerns raised when a catheter became hard to withdraw a CVC after cardiac surgery.

## 2. Case History

A 65-year-old female patient who was suffering from severe mitral valve stenosis (area of 0.6 cm^2^) plus coronary artery disease underwent mitral valve replacement and 2-vessel coronary artery bypass graft (CABG) surgeries simultaneously at Shahid Rajaee hospital of Iran, University of Medical Sciences (Tehran, Iran) in May 2009.

At preanesthetic evaluations, she had history of diabetes and hypertension. After induction of general anesthesia and intubation, through the right internal jugular vein, a 20-cm long, 7.5 Fr multilumen central venous catheter was inserted by a Seldinger's technique. Its proper placement in right atrium and SVC junction was confirmed by appearance of waveforms on monitors; moreover, its patency was checked by free aspiration of blood from all three lumens.

After completing surgery, fluid could not be flushed, and the patient was uneventfully transferred to cardiac surgery ICU, where CVC was reconfirmed to be patent. Initial X-ray did not show any tortuousness or knicking of CVC.

The entrapment of the CVC remained unnoticed until the time of removal on the third postoperative day when attempts for removal by gentle traction failed. Blood could not be aspirated, and fluid could not be flushed from the distal port of the triple lumen catheter. Repeating X-ray illustrated its straight way from entry point to its 3 cm distal end point at which it showed a tilt.

**Figure 1:**
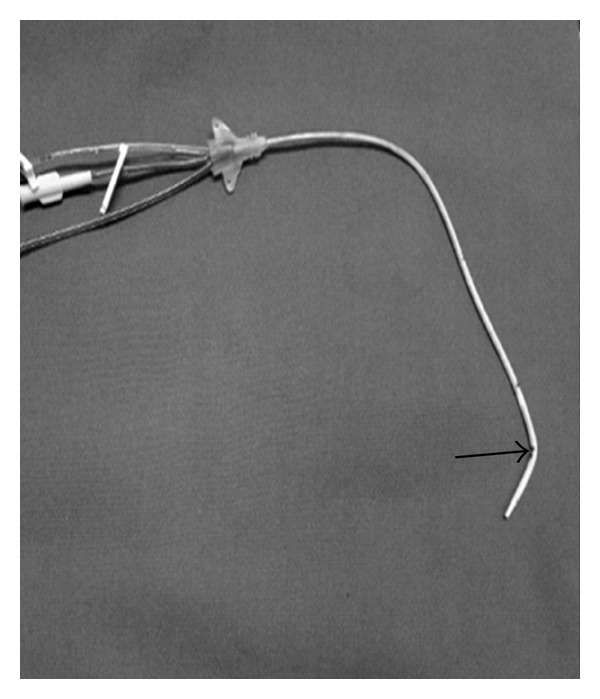
Photograph of left CVC after removal. Note the distal tip that became tilted (Arrow).

With diagnosis of possible bending of the catheter, the patient underwent fluoroscopy. Dye injection under fluoroscopy confirmed the initial findings (Figure 1). After confirmation of opening the lumen, a trial made to pass a guide wire through main port. It was passed with minimal resistance through its distal end, but again resistance appeared removing the catheter from the inserted state. It led to a high index of suspicion, for CVC being sutured to the heart.

**Figure 2:**
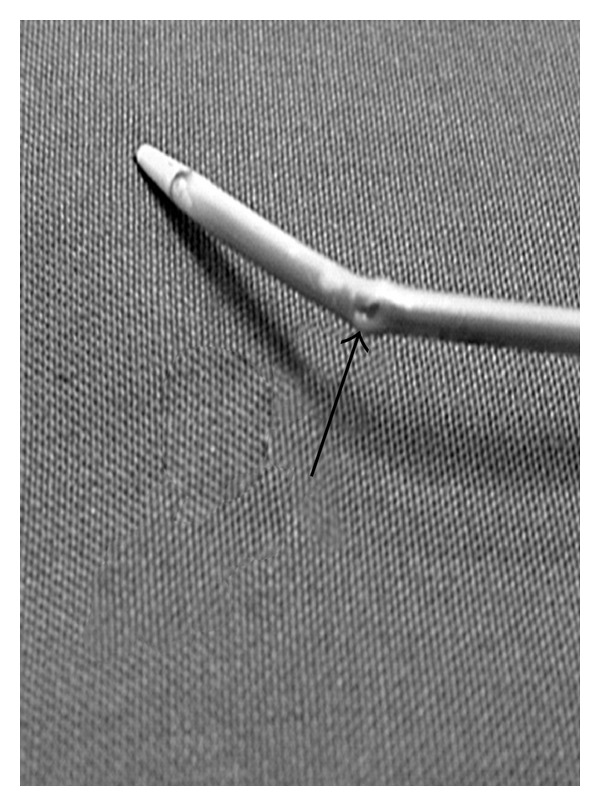
The distal section of CVC. It is possible to see the hole made by purse-string suture (Arrow).

Surgical exploration with sternotomy under general anesthesia was performed. A reinforcement stitch was first placed in the upper end of right atrium to prevent excessive bleeding. Then the suture entrapping the CVC was freed. It was the purse-string suture at the venous cannulation site of CPB. Then CVC could be easily pulled out (Figure 2). It was possible to see the hole made by purse-string suture.

## 3. Discussion

Stuck CVC is an unusual complication. Causes of stuck CVC include shearing or fracturing of the CVC, fibrin sheath formation, infectious process, thrombosis, venospasm, catheter looping, and knotting [4, 6, 7].

During open heart surgery, the CVC tends to lie against the lateral or the posterior wall of the right atrium, where it may be caught by a suture in the during venous cannulation for CPB [8]; however, suturing of a CVC or pulmonary artery catheters (PACs) to heart and vessels by cardiac sutures has rarely been reported [2, 8]. Although the literature contains few reports of other types of CVC entrapments, we believe that our case to be one of the very few reports of CVC was sutured surgically to the wall of the right atrium while doing the purse suture for inserting the inferior vena cava cannula prior to CPB and necessitated reexploration of the chest to remove it.

Kaplan et al. [9] surveyed 10 cases of PAC entrapment complications, all of which involved valvular replacement surgeries. Huang et al. [8] similarly reported entrapment of a Swan-Ganz catheter in the purse-string suture in a patient undergoing aortic valve replacement. Our case was also during valve replacement CABG surgery.

## 4. Diagnosis

Several diagnostic methods could be used to diagnose the suture entrapment. In most of pulmonary artery catheter entrapment reports, diagnosis was suspected when resistance was felt while attempting to withdraw this venting catheter, and confirmed by fluoroscopy postoperatively [10–14]. As reported in our case, acute angulation of the catheter on chest radiograph is an important diagnostic sign, and it confirmed the possibility of suture entrapment [13, 15]. Transesophageal echocardiogram has been used as a very useful tool for the diagnosis of suture entrapment of a Swan-Ganz catheter during open heart [16–18].

## 5. Management

Various methods of dealing with stuck catheters have been explored depending upon the cause of entrapment. As the gentle trial and passing a guide wire failed to move the CVC, it was decided to remove it surgically. Since there was a risk of opening the suture line or of a rupture while trying to remove the catheter by nonsurgical methods, we preferred the surgical method. Atrial wall laceration could occur if an encircled suture removed by forceful traction [19]. Most similar cases required a repeat sternotomy with or without CPB [9, 20].

## 6. Prevention

To prevent this complication is difficult due to its very low incidence, and care should be taken while closing the cannulation site of the right atrium. We wonder whether it would be possible for anesthesiologists or surgeons to prevent similar from happening. A possible solution includes simply pulling CVC for few centimeters by anesthesiologist after purse-string sutures at the venous cannulation site are tied. If any resistance is felt, the suture can be gently loosened and the catheter pulled out of the suture line into the cephalic portion of the superior vena cava. Yet, if the complication is realized postoperatively, it must be managed surgically, as described in our case.

Some authors recommend ensuring catheter mobility before the chest is closed preventing PAC entrapment [9, 14].

## 7. Conclusion

This report, besides other reports, shows that when resistance was met on a CVC withdrawal after open heart surgery, we should never withdraw it forcefully and blindly. One should consider inadvertent entrapment of CVC by a suture as the possible cause although it is an unusual complication. This complication can probably be avoided by care taken by the surgeon at the time of closure of the right atrium not to leave CVC in the sutures and after completion of the sutures, by anesthesiologist by partial withdrawal of the CVC at the time of coming off CPB, or at least before chest closure.
